# A meta-analysis of anti-interleukin-13 monoclonal antibodies for uncontrolled asthma

**DOI:** 10.1371/journal.pone.0211790

**Published:** 2019-01-31

**Authors:** Hang Li, Kai Wang, Huiting Huang, Wenbin Cheng, Xiaohong Liu

**Affiliations:** 1 The First Clinical College, Guangzhou University of Chinese Medicine, Guangzhou, Guangdong, China; 2 Department of Respiratory Medicine, Shenzhen Bao’an Traditional Chinese Medicine Hospital (Group), Shenzhen, Guangdong, China; 3 Department of Respiratory Medicine, The First Affiliated Hospital of Guangzhou University of Chinese Medicine, Guangzhou, Guangdong, China; Hvidovre Hospital, DENMARK

## Abstract

More and more clinical trials have tried to assess the clinical benefit of anti-interleukin (IL)-13 monoclonal antibodies for uncontrolled asthma. The aim of this study is to evaluate the efficacy and safety of anti-IL-13 monoclonal antibodies for uncontrolled asthma. Major databases were searched for randomized controlled trials comparing the anti-IL-13 treatment and a placebo in uncontrolled asthma. Outcomes, including asthma exacerbation rate, forced expiratory volume in 1 second (FEV_1_), Asthma Quality of Life Questionnaire (AQLQ) scores, rescue medication use, and adverse events were extracted from included studies for systematic review and meta-analysis. Five studies involving 3476 patients and two anti-IL-13 antibodies (lebrikizumab and tralokinumab) were included in this meta-analysis. Compared to the placebo, anti-IL-13 treatments were associated with significant improvement in asthma exacerbation, FEV_1_ and AQLQ scores, and reduction in rescue medication use. Adverse events and serious adverse events were similar between two groups. Subgroup analysis showed patients with high periostin level had a lower risk of asthma exacerbation after receiving anti-IL-13 treatment. Our study suggests that anti-IL-13 monoclonal antibodies could improve the management of uncontrolled asthma. Periostin may be a good biomarker to detect the specific subgroup who could get better response to anti-IL-13 treatments. In view of blocking IL-13 alone is possibly not enough to achieve asthma control because of the overlapping pathophysiological roles of IL-13/IL-4 in inflammatory pathways, combined blocking of IL-13 and IL-4 with monoclonal antibodies may be more encouraging.

## Introduction

With the clinical use of inhaled corticosteroid (ICS) and long-acting inhaled bronchodilators, the symptoms of most of asthma patients can be well controlled. However, despite regular treatment in current guidelines, there are still about 40% of asthma patients still have trouble controlling their symptoms[1, 2]. Unsatisfactory control of symptom is closely related to an increased risk of asthma exacerbation and mortality—impairing patients’ life quality and accounting for a high financial burden[3, 4]. Thus, it is quite necessary to improve management and control of asthma. Some novel therapeutic options for uncontrolled asthma have been used in clinic or undergoing clinical trials. Some monoclonal antibodies, such as anti-Interleukin (IL)-5 (benralizumab and mepolizumab) and anti-IgE (omalizumab), have showed clinical efficacy in treating severe refractory asthma by inhibiting T helper 2 (Th2) cytokine-mediated inflammation response[5, 6]. They have been recommended to be add-on treatments for asthma in Global Initiative for Asthma (GINA) guideline[7].

IL-13, another cytokine relative to Th2 lymphocyte-mediated inflammation, was involved in pathology of asthma, including recruitment of eosinophils and basophils, mucus production, goblet cell differentiation and IgE synthesis[8–11]. Several humanized monoclonal antibodies to IL-13, including anrukinzumab, lebrikizumab and tralokinumab, are currently under clinical evaluation[12, 13]. Since IL-13 is a crucial regulator of refractory asthma, it has been identified as a potential therapeutic target for patients with uncontrolled asthma[14, 15]. Many randomized controlled trials (RCTs) have showed promising results when treating uncontrolled asthma with anti-IL-13 monoclonal antibodies. However, the effects of these treatments on the rate of asthma exacerbation and quality of life lack of uniformity. In addition, the value of outcomes from individual clinical trial is relatively limited. Pooled analysis of these individual studies can provide comprehensive information to objectively evaluate the potentiality of anti-IL-13 antibodies for the management of uncontrolled asthma. Thus, we carried out a meta-analysis of RCTs to evaluate efficacy (asthma exacerbation, lung function, life quality and rescue medication use) and safety (adverse events) of anti-IL-13 antibodies for uncontrolled asthma.

## Methods

### Search strategy

This meta-analysis was registered at International Prospective Register of Systematic Reviews (registration number: CRD42018081649) and performed according to the Preferred Reporting Items for Systematic Reviews and Meta-Analyses (PRISMA) Statement. [16] We selected published studies by searching PubMed, Cochrane, EMBASE and ClinicalTrials.gov databases (between January 1, 1950 and November 1, 2017). A literature search was conducted using a combination of the term “uncontrolled asthma” and the following text words: “anti-IL-13” or “anti-Interleukin-13” or “lebrikizumab” or “tralokinumab” or “GSK679586”. The search details used for PubMed was as follows: uncontrolled [All Fields] AND ("asthma"[MeSH Terms] OR "asthma"[All Fields])) AND ((((anti-IL-13[Text Word] OR anti-Interleukin-13[Text Word]) OR lebrikizumab[Text Word]) OR tralokinumab[Text Word]) OR GSK679586[Text Word]). No language restrictions were applied. The reference lists of key papers were also searched manually.

### Eligibility criteria

The inclusion criteria were: (1) adults patients (≥ 18 years) with poorly controlled asthma despite ICS or ICS plus long acting beta-agonist(LABA) use; and (2) randomized controlled trials; (3) comparing interventions between anti-IL-13 monoclonal antibodies and placebo; (4) reporting any outcomes of interest: changes in forced expiratory volume in 1 second (FEV_1_), exacerbation of asthma, Asthma Quality of Life Questionnaire (AQLQ) scores, rescue medication use or adverse events. We excluded non-RCT, observational or retrospective studies. HL and KW independently screened all studies according to the eligibility criteria. Disagreements in opinion about inclusion were discussed and resolved by consensus or arbitration of a third investigator(XHL).

### Data extraction

HL and WBC independently reviewed full-text and extracted related data from eligible studies which satisfied the selection criteria; disagreements were resolved by a third investigator (HHT). The primary outcomes were change in rate of asthma exacerbation and FEV_1_ between baseline and end of intervention. Asthma exacerbation was defined as worsening asthma symptoms requiring high-dose of inhaled corticosteroids therapy or oral corticosteroids therapy or the need for hospitalization. The secondary outcomes were AQLQ(S), rescue medication use and adverse events.

### Statistical analysis

We focused on assessing the effect of intervention on five outcomes: FEV_1_, rate of asthma exacerbation, AQLQ(S), use of rescue medication, and adverse events. Rate of asthma exacerbation, FEV_1_, AQLQ(S) and use of rescue medication were analyzed as continuous variables, and they were reported in changes from end of intervention to baseline. We calculated the mean differences (MD) and 95% CI of them between intervention groups. If one study had more than one intervention group for anti-IL-13 treatment, we combined all intervention groups into one group according to Cochrane handbook. Adverse events and serious adverse events were analyzed as dichotomous data, and calculated in relative risk (RR) and 95% CI. Heterogeneity was evaluated by the I^2^ test, and I^2^>50% and P-value<0.05 was regarded as significant heterogeneity. Fixed-effects model was used if there was no significant heterogeneity. Random-effects model would be used if there was significant heterogeneity. Subgroup analyses were conducted based off of serum periostin level and different medicines. The risk of bias of included trials was evaluated by recommended tools from Cochrane Handbook. We did sensitivity analysis for overall effect by sequentially excluding every single trial. Review Manager Software (Version 5.3) was used for statistical analyses.

## Results

### Study characteristics

**Fig 1:**
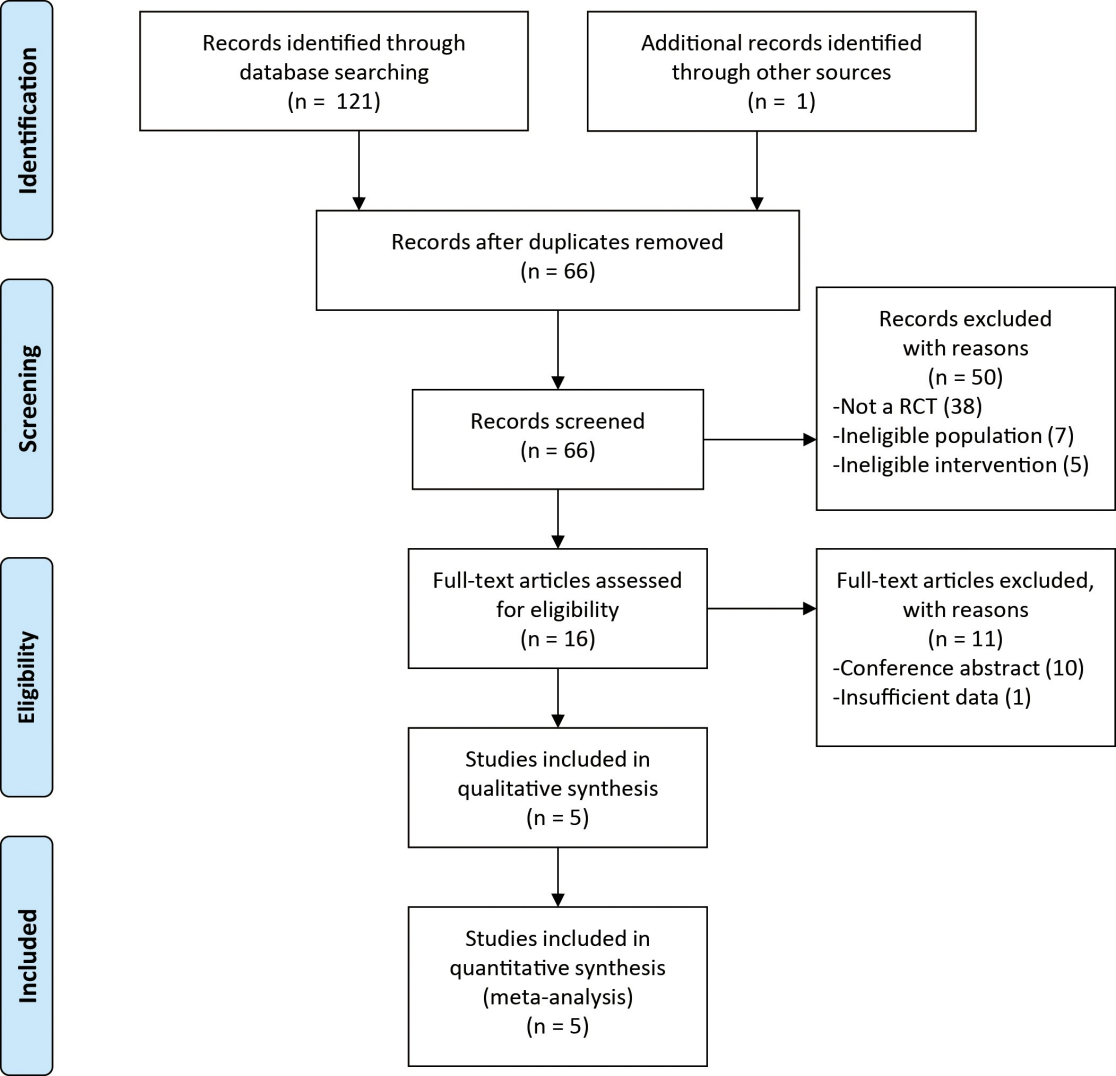
Flow chart of study selection process.

We identified 121 studies: 22 from PubMed, 22 from Cochrane, 66 from EMBASE, 11 from ClinicalTrials.gov, and 1 from manual search. After duplicates removed and title/abstract and full-text screening, 5 studies[17–21] (with data for 3476 participants) were included in our analysis (Fig 1). Table 1 describes the characteristics of included RCTs. They were published between 2011 and 2016. Three and two trials used lebrikizumab and tralokinumab, respectively. Administered doses and frequency were different between different trials. Treatment duration ranged from 12 weeks to 52 weeks. Patients had mean baseline FEV_1_ (%pred) of 62.3 (10.5). A 2016 study[21] published by Hanania et al. was regarded as two independent trials when assessing rate of asthma exacerbation, FEV_1_, AQLQ(S), and rescue medication use because of following reasons: (1) they were two replicate and independent trials; (2) the participants (2148) in this study accounted for nearly 60% of total participants (3476) in all studies; (3) complete and independent data were available in two trials.

**Table 1:** Characteristics of included studies.

Source	Study design	No. of subjects	No. of female	Mean age (years)	Drug	Dose	Treatment duration (weeks)	Baseline FEV_1_(%pred) mean (SD)
Corren 2011 [18]	double-blind, parallel-group, multicenter RCT	219	143(66%)	44.0	lebrikizumab	250 mg, once a month	24	65(11)
Piper 2013 [20]	double-blind, parallel-group, multicentre RCT	194	116(59.8%)	47.4	tralokinumab	150, 300 or 600 mg every 2 weeks	12	61.2(12.3)
Brightling 2015 [17]	double-blind, parallel-group, multicentre RCT	452	297(65.7%)	50.2	tralokinumab	300 mg, every 2 weeks or every 2 weeks for 12 weeks then every 4 weeks	48 or 50	68.5(18.2)
Hanania 2015 [19]	replicate, multicentre, double-blind RCT	463	275(59.4%)	48.4	lebrikizumab	37.5, 125,250 mg every 4 weeks	24(median)	62.2(10.4)
Hanania 2016 [21]	replicate, double-blind, multicentre, multinational RCT	2148	1371(63.8%)	50.7	lebrikizumab	37.5 mg or 125 mg, every 4 weeks	52	60.9(10.5)

RCT = randomized controlled trial. FEV_1_ = forced expiratory volume in 1 second. FEV_1_ (%pred) = FEV_1_ of predicted value.

### Primary outcomes

#### Rate of asthma exacerbation

**Fig 2:**
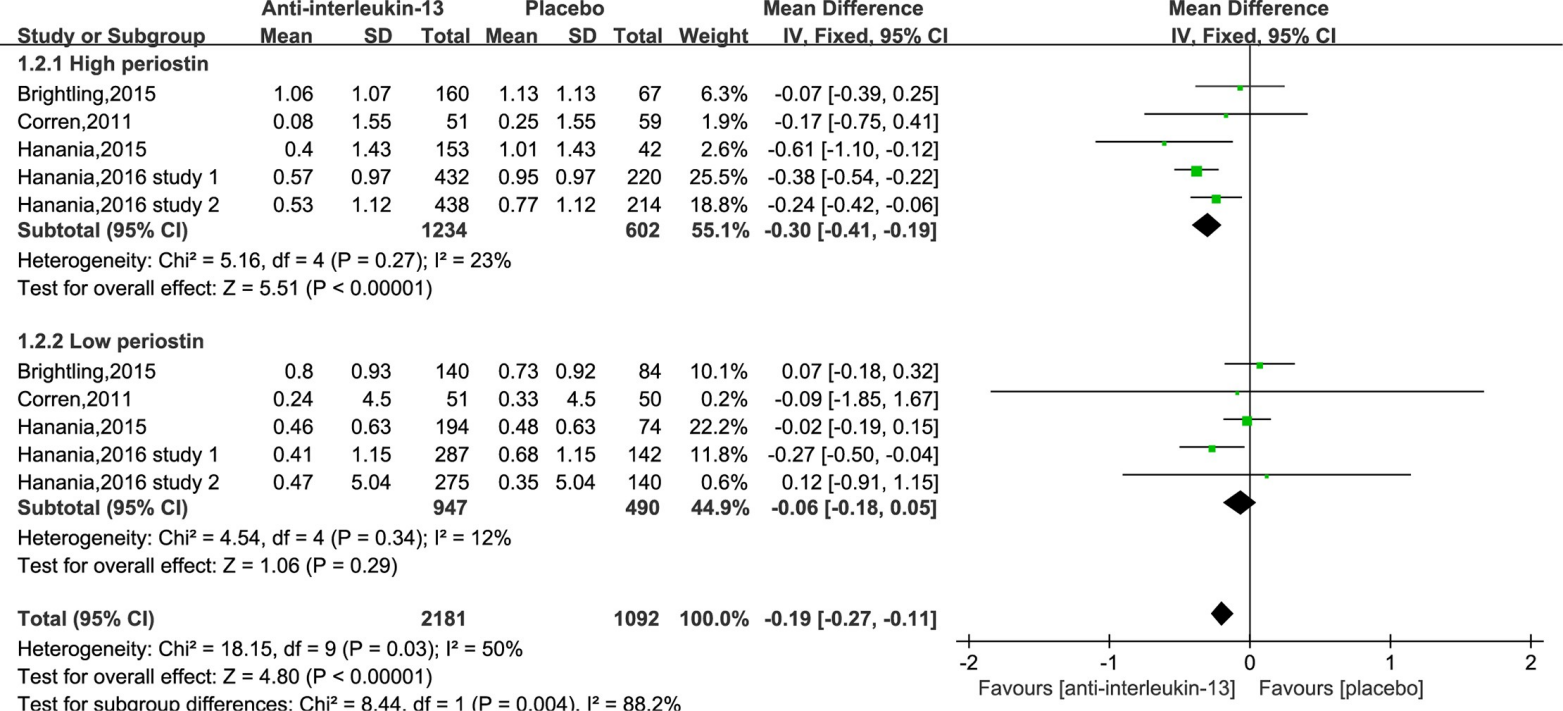
Forest plot comparing anti-IL-13 and placebo with rate of asthma exacerbation.

Asthma exacerbation data were available in five trials[17–19, 21]. Pooled analysis showed a significant reduction of risk in asthma exacerbation when participants were treated with lebrikizumab and tralokinumab(MD = -0.19, 95%CI: -0.27–0.11, P <0.001), with statistically significant between-study heterogeneity(I^2^ = 50%, P = 0.03) (Fig 2). In the further sensitivity analysis, we assessed the overall effect on rate of asthma exacerbation by sequentially excluding one trial. After individually excluding two trials, no significant difference in asthma exacerbation was showed between anti-IL-13 treatment and placebo group. Subgroup analysis showed patients with high periostin level (>50 ng/ml) had a lower risk of asthma exacerbation after receiving anti-IL-13 treatment (MD = -0.30, 95%CI: -0.41–0.19, P<0.001). No significant heterogeneity was found (I^2^ = 23%, P = 0.27). However, we saw no treatment benefit in patients with low periostin level (MD = -0.06, 95%CI: -0.18–0.05, P = 0.34).

#### FEV_1_

**Fig 3:**
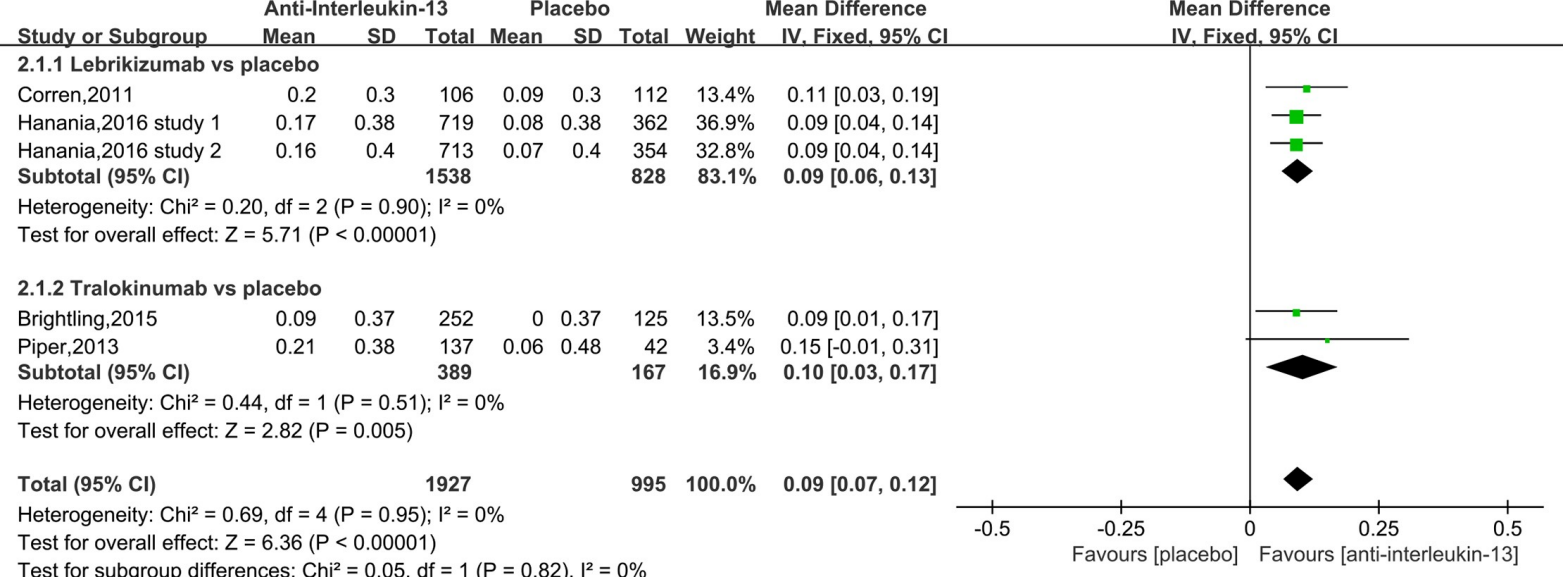
Forest plot comparing anti-IL-13 and placebo with FEV_1_.

Data on FEV_1_ were available in five trials. ^17, 18, 20, 21^ Our results showed anti-IL-13 treatment increased patients’ FEV_1_ compared to the placebo (MD = 0.09, 95%CI: 0.07–0.12, P <0.001), with no significant heterogeneity (I^2^ = 0%, P = 0.95) (Fig 3). Subgroup analysis comparing lebrikizumab treatment to the placebo showed an increase in FEV_1_ in favors of lebrikizumab (MD = 0.09, 95%CI: 0.06–0.13, P <0.001), with no significant heterogeneity (I^2^ = 0%, P = 0.9). Subgroup analysis comparing tralokinumab treatment to the placebo also showed similar results (MD = 0.10, 95%CI: 0.03–0.17, P = 0.005), with no significant heterogeneity (I^2^ = 0%, P = 0.51). There is no significant difference between the two subgroups.

### Secondary outcomes

#### AQLQ(S)

**Fig 4:**
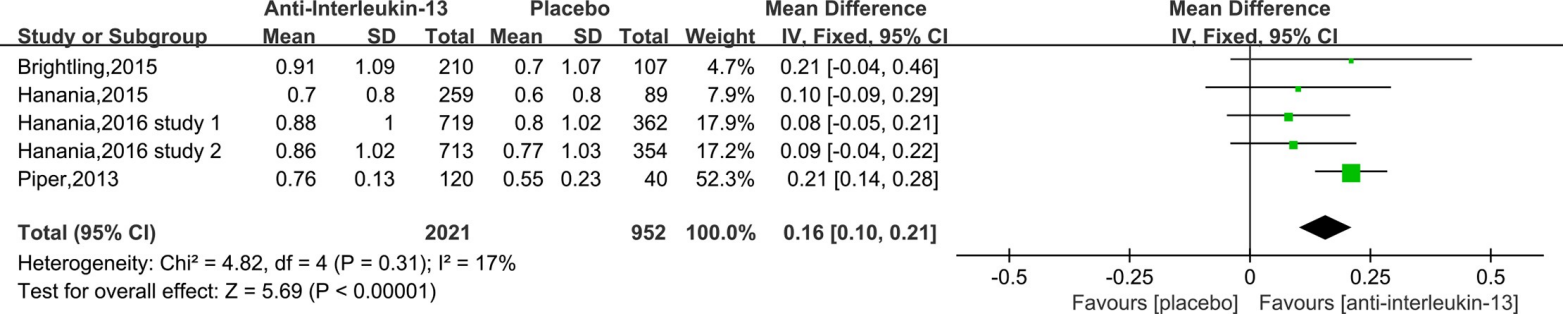
Forest plot comparing anti-IL-13 and placebo with AQLQ scores.

Pooled analysis of the 5 studies[17, 19–21] that assessed change in AQLQ(S) from the end of intervention to baseline showed anti-IL-13 treatment was associated with greater improvement in AQLQ(S) (MD = 0.16, 95%CI: 0.10–0.21, P <0.00001), with no significant heterogeneity (I^2^ = 17%, P = 0.31) (Fig 4).

#### Rescue medication use

**Fig 5:**
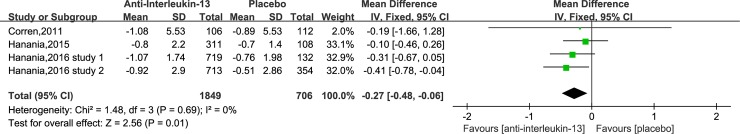
Forest plot comparing anti-IL-13 and placebo with rescue medication use.

Four studies[18, 19, 21] evaluated the effect of anti-IL-13 treatments verse the placebo on rescue medication use. Pooled analysis of these studies showed a significant decrease in rescue medication use in anti-IL-13 group compared to the placebo group (MD = -0.27, 95%CI: -0.48–0.06, P = 0.01), with no significant heterogeneity (I^2^ = 0%, P = 0.69) (Fig 5).

#### Adverse events

**Fig 6:**
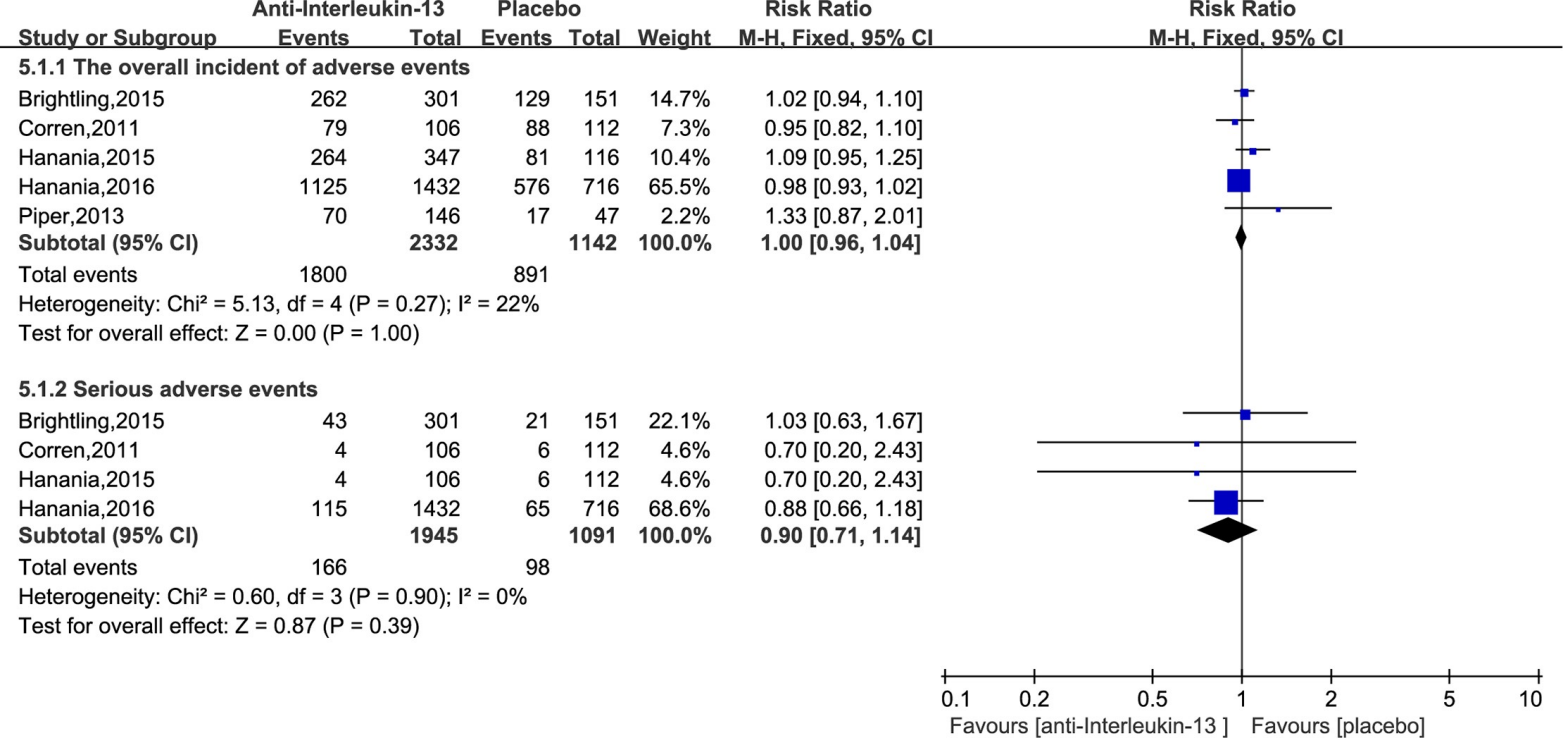
Forest plot comparing anti-IL-13 and placebo with adverse events and serious adverse events.

Five studies[17–21] reported the effect of anti-IL-13 antibodies verses the placebo on adverse events. Pooled RR was 1.00 (95% CI: 0.96–1.04), which meant no significant difference in adverse events between anti-IL-13 and placebo groups, with no significant heterogeneity (I^2^ = 22%, P = 0.27). Four trials[17–19, 21] also assessed the effect of anti-IL-13 antibodies verses the placebo on serious adverse events. No significant difference was found between anti-IL-13 and placebo group (RR = 0.90, 95%CI = 0.71–1.14), with no significant heterogeneity (I2 = 0%, P = 0.9). These results showed that anti-IL-13 treatment was well tolerated. (Fig 6)

### Risk of bias

**Fig 7:**
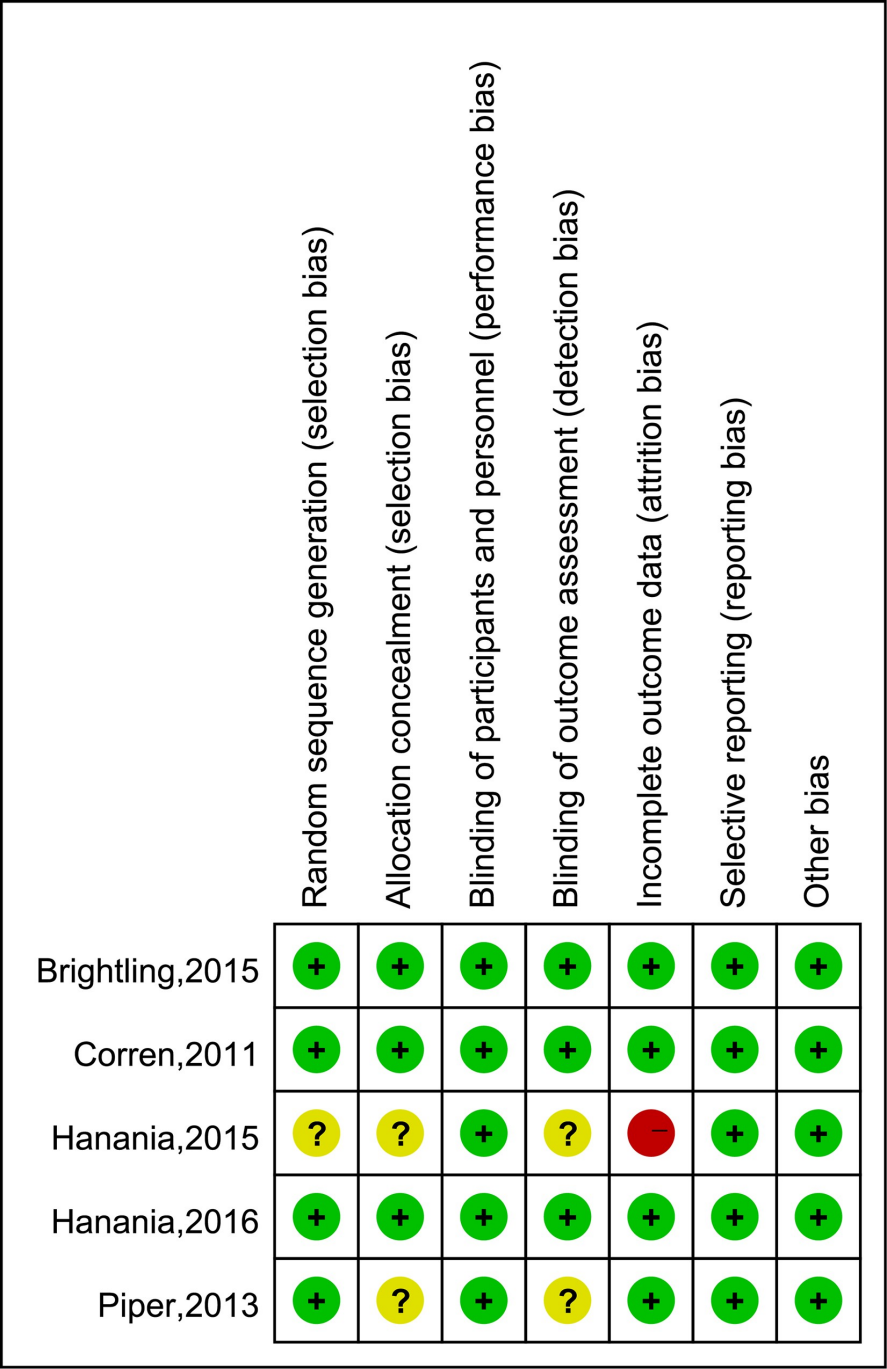
Risk of bias summary.

Risk of bias of each included study is evaluated and summarized in Fig 7. Four RCTs[17, 18, 20, 21] reported adequate randomization sequence generation, but one trial[19] didn’t specify that. Two trials didn’t mention how they did the allocation concealment[19, 20]. All trials were described as double-blinded. Two trials did not specify whether outcome assessment was masked to treatment allocation[19, 20]. One study may have attrition bias due to study protocol modification[19].

## Discussion

Asthma is a heterogeneous disease with different phenotypes, clinical features, and responses to treatments [22, 23]. Specific molecular patterns in different phenotypes of asthma could be possible target of treatment[24]. Some patients with uncontrolled asthma still have a high level of IL-13 even when treated by inhaled or systemic glucocorticoid[14], which is consistent with the hypothesis that IL-13 can contribute to steroid resistance[25, 26]. A systematic review published in 2016 assessed the efficacy of anti-IL-13 antibodies in patients with mild to severe asthma[27]. It showed anti-IL-13 antibodies could improve peak expiratory flow, decrease FeNO and asthma exacerbation. However, that systematic review included asthma patients in different severity from mild to severe, and it didn’t assess the possibility of periostin level as biomarker for anti-IL-13 treatments. Our meta-analysis focused on studying patients with uncontrolled asthma, because mild asthma could be comparatively easier to be controlled by common therapies. In the 2017 GINA guideline, anti-IL-13 therapies haven’t been recommended to be add-on treatments for asthma. Our meta-analysis investigated the potential of anti-IL-13 to be an addition on current asthma controller therapies in patients with uncontrolled asthma.

In clinical trials or in clinical practice, assessment of asthma control needs multiple domains[28]. Our findings assessed an overall beneficial effect on anti-IL-13 treatment. Asthma exacerbation is closely associated with mortality, and reducing asthma exacerbation rate is a central goal in the management of asthma[29, 30]. Our results showed that reduced asthma exacerbation rate was significantly associated with high periostin level (>50 ng/ml) in patients’ serum. Periostin, a matricelllular protein secreted by bronchial epithelial cells, is associated with IL-13 activity in the lung, and served as biomarker of eosinophil airway inflammation[31, 32]. Although the data are paradoxical with respect to its linking with Th2 response[33, 34], this finding suggests that periostin could be helpful to detect the specific subgroup who could get better response to anti-IL-13 interventions. The intervention medicines included in our systematic review are lebrikizumab and tralokinumab. Subgroup analysis showed they were associated with improvement of lung function. In terms of change in FEV_1,_ no significant difference was found between lebrikizumab and tralokinumab. In addition, reduction in the use of rescue medication is consistent with the decrease in rate of asthma exacerbation.

Our results showed adverse events and serious adverse events were similar between the anti-IL-13 group and placebo group, which meant anti-IL-13 treatments were well tolerated. However, there were some adverse events related to anti-IL-13 treatments that still need to be recognized. One study reported musculoskeletal events were more common in subjects who received lebrikizumab than the placebo (13.2% vs. 5.4%)[18]. Compared to the placebo, lebrikizumab and tralokinumab were easier to cause injection site reactions[19, 20]. Risk of elevating level of eosinophils was observed in Hanania’s study[21]. Diarrhoea (3.4%) and urinary-related adverse events including bacteriuria (5.5%), urinary tract infections (4.1%) and crystalluria (4.3%) were reported only in the tralokinumab group[20].

As mentioned in recent report of Polyxeni Ntontsi, et al, it must be noted that blocking IL-13 alone is possibly not enough to achieve asthma control[35]. During asthmatic inflammation, IL-4 and IL-13 bind to specific receptor expressed in diverse inflammatory cells. However, circulating IL-13 binds to complex receptors and then activates and recruits IL-4 receptors. Therefore, patients may benefit more from combined blocking of IL-4 and IL-13 with monoclonal antibodies because of the overlapping pathophysiological roles of IL-13/IL-4 in asthma.

Observing difference in efficacy between different doses of lebrikizumab and tralokinumab could add to our knowledge and understanding of the therapeutical potential of these monoclonal antibodies. For lebrikizumab, there were 3 doses (37.5, 125 and 250mg) in phase 2 LUTE/VERSE studies[19] and 2 doses (37.5 and 125 mg) in phase 3 LAVOLTA Ⅰand Ⅱstudies[21]. Overall, no distinct dose–response on exacerbation rates was observed for lebrikizumab. Conversely, the highest exacerbation rate reduction was noted in the lowest dose group (37·5 mg). However, FEV_1_ might be more sensitive for doses since 125 mg dose provided a bit better improvement in FEV_1_ compared with 37.5 mg dose. For tralokinumab, there were 3 doses (150, 300 and 600mg) used in Piper’s study[20]. The percentage of FEV_1_ rising from baseline ranged from 8.1% (150 mg) to 16.1% (600 mg) suggesting that tralokinumab might deliver a clinical dose-response. In Brightling’s study[17], single dose of tralokinumab (300 mg) was administrated every 2 weeks or every 4 weeks. Compared with placebo group, there was no significant difference in the increase of FEV_1_ given tralokinumab every 4 weeks suggesting that taking it every two weeks is the most effective.

This meta-analysis has some limitations. Firstly, the number of included trials is small. More RCTs should be performed to offer more evidences and lead to a stronger conclusion. In addition, due to limited included trials, publication bias could not be well assessed by Egger’s test and Begg’s funnel. Secondly, in this meta-analysis, two or three intervention groups were combined into one single intervention group, which ignored different administration doses, and made it hard to identify the optimal dose. Thirdly, there were some differences in the definition of uncontrolled asthma between different studies. That may lead to heterogeneity in the inclusion criteria for subjects. However, the similarity in the definition of uncontrolled asthma in different studies is that Asthma Control Questionnaire score (ACQ-5 or ACQ-6) is 1.5 or higher at screening and randomization. Fourth, as the number of patients included in the study by Hanania et al 2016 is much more than other studies, some potential limitation/ bias of this particular study may be caused.

As we know, this is the first systematic review assessing efficacy and safety of anti-IL-13 monoclonal antibodies for uncontrolled asthma. Our study shows that targeting IL-13 therapies could be beneficial for asthmatic patients in terms of pulmonary function and exacerbation rate. In addition, periostin may be a good biomarker to detect the specific subgroup who could get better response to anti-IL-13 treatments. In view of blocking IL-13 alone is possibly not enough to achieve asthma control because of the overlapping pathophysiological roles of IL-13/IL-4 in inflammatory pathways, combined blocking of IL-13 and IL-4 with monoclonal antibodies may be more encouraging.

## Supporting information

S1 Checklist PRISMA checklist. (DOC)
